# Transcriptional Knock-down of *mstn* Encoding Myostatin Improves Muscle Quality of Nile Tilapia (*Oreochromis niloticus*)

**DOI:** 10.1007/s10126-023-10252-1

**Published:** 2023-09-27

**Authors:** Qingchun Wang, Yue Yan, Yifan Tao, Siqi Lu, Pao Xu, Jun Qiang

**Affiliations:** 1 Wuxi Fisheries College, Nanjing Agricultural University, Wuxi, 214081 China; 2 Key Laboratory of Freshwater Fishes and Germplasm Resources Utilization, Ministry of Agriculture, Freshwater Fisheries Research Center, Chinese Academy of Fishery Sciences, Wuxi, 214081 China

**Keywords:** Antisense RNA, *mstn* transcription inhibition, Muscle development, Lipid metabolism

## Abstract

Myostatin (encoded by *mstn*) negatively regulates skeletal muscle mass and affects lipid metabolism. To explore the regulatory effects of *mstn* on muscle development and lipid metabolism in Nile tilapia (*Oreochromis niloticus*), we used antisense RNA to transcriptionally knock-down *mstn*. At 180 days, the body weight and body length were significantly higher in the *mstn-*knock-down group than in the control group (*p* < 0.05). Additionally, fish with *mstn*-knock-down exhibited myofiber hyperplasia but not hypertrophy. Oil red O staining revealed a remarkable increase in the area of lipid droplets in muscle in the *mstn*-knockdown group (*p* < 0.05). Nutrient composition analyses of muscle tissue showed that the crude fat content was significantly increased in the *mstn*-knock-down group (*p* < 0.05). The contents of saturated fatty acids, monounsaturated fatty acids, and polyunsaturated fatty acids were all significantly increased in the *mstn*-knock-down group (*p* < 0.05). Comparative transcriptome analyses revealed 2420 significant differentially expressed genes between the *mstn*-knock-down group and the control group. KEGG analysis indicates that disruptions to fatty acid degradation, glycerolipid metabolism, and the PPAR signaling pathway affect muscle development and lipid metabolism in *mstn*-knock-down Nile tilapia: *acaa2*, *eci1*, and *lepr* were remarkably up-regulated, and *acadvl*, *lpl*, *foxo3*, *myod1*, *myog*, and *myf5* were significantly down-regulated (*p* < 0.05). These results show that knock-down of *mstn* results in abnormal lipid metabolism, acceleration of skeletal muscle development, and increased adipogenesis and weight gain in Nile tilapia.

**Supplementary Information:**

The online version contains supplementary material available at 10.1007/s10126-023-10252-1.

## Introduction

Skeletal muscle regulates many physiological activities and allows the body to maintain various poses and generate fluid power. The *mstn* gene, which encodes myostatin, is highly expressed in skeletal muscle and adipose tissue (Roberts and Goetz 2001). Similar to *mstn* genes in other vertebrates, *mstn* genes in teleosts encode a glycoprotein and are widely expressed throughout the body (Xu et al. 2003). The sequence of *mstn* is highly conserved among fish species, and *mstn*-deficient fish exhibit a double-muscled phenotype (Chisada et al. 2011). In fish, myostatin not only inhibits skeletal muscle growth, but also maintains homeostasis of tissue growth, functions in reproductive tissue, and participates in the regulation of osmotic pressure (Rodgers and Weber 2001). Myostatin is also involved in the regulation of fat accumulation (Gao et al. 2019). In the pig (*Susscrofa domestica*) model, *mstn*-deficient pigs show increased muscle mass, enhance oxidation of adipose tissue, and decrease fat deposition (Xuan et al. 2022). Disruption of *mstn* through CRISPR/Cas9 in blunt snout bream (*Megalobrama amblycephala*) was shown to result in increased muscle mass (Sun et al. 2020). Whereas the effects of *mstn* on muscle quality are well understood in mammals, but little is known about its effects in Nile tilapia.

Intramuscular fat (IMF) plays a role in connecting skeletal muscles, and its content affects meat quality. The IMF is distributed in skeletal muscle and is visible as white spots or stripes between muscle fibers. The IMF content affects various quality traits of fish muscle, because within a certain range, a higher IMF content improves the sensory quality and shape of muscle. Thus, the fat content in muscle is an important fish quality index. The skeletal muscle is the main edible part of fish. The growth of tilapia is focused on striated skeletal muscle, which accounts for 40–75% of total body weight (Zhou et al. 2023). Because white adipose tissue makes up more than 90% of most muscle tissue, teleost fish gain weight mainly because of changes in the fat content of muscle tissue (Alami-Durante et al. 2010). In white adipose tissue, lipid droplets fuse to store energy for later use (Walden et al. 2012). Triglycerides (TG) in adipose tissue are stored in lipid droplets, so there are small fat droplets in skeletal muscle (Yang et al. 2019). Muscle quality and nutrient content directly affect the sensory quality of meat from cultured fish, and this is an important economic index in the aquaculture industry. Inhibition of *mstn* expression may lead to the loss of other important biological functions, which may adversely affect the health of gene-knocked-down fish. In this study, we knocked-down *mstn* in Nile tilapia to determine its effects on muscle development and lipid metabolism and the key metabolic processes involved in its regulation.

The rapid growth and strong stress resistance of tilapia make it an important aquaculture species. The wide salinity tolerance of tilapia enables it to survive in freshwater, brackish water, and hypersaline water (Yan et al. 2013). This not only renders tilapia suitable for freshwater aquaculture but also endows it with significant potential for mariculture. The traditional breeding model, however, is characterized by a lengthy cycle and low efficiency. Therefore, it is imperative to explore genetic approaches for enhancing the growth rate and meat yield of tilapia. Our laboratory has successfully used antisense RNA technology for gene editing of Nile tilapia (Cao et al. 2022; Qiang et al 2022; Yan et al. 2022). This method is convenient, and the phenotype of the offspring is stable (Cao et al. 2022). In this study, we designed antisense RNA sequences and introduced them into the ovum through the micropore fertilization pore to construct a model of knocked-down *mstn*. The growth performance of the fish was determined; changes in skeletal muscle were detected by histological analysis, and the nutritional composition of muscle tissue was analyzed. We detected the *mstn* transcript level and myostatin protein (MSTN) expression level, conducted histological analysis and nutritional analysis of muscle tissue, and identified the genes and pathways affected by *mstn* knock-down. The overall aim of this study was to explore the how muscle development and lipid metabolism are regulated in Nile tilapia. Our results provide a theoretical basis for breeding new tilapia lines with high meat quality in the future, aiming to enhance both marine and freshwater tilapia aquaculture production.

## Materials and Methods

### Ethics Statement

The research was approved by the Ethics Committee of the Freshwater Fisheries Research Center of Chinese Academy of Fishery Sciences (FFRC, Wuxi, China). Sampling was conducted according to the Guide for the Care and Use of Experimental Animals in China.

### Acclimation of Fish Before Experiment

Brood stock were taken from FFRC and raised in indoor recirculating tanks (temperature 27–29 °C, dissolved oxygen > 6 mg/L, pH 7.4–7.8). The fish were fed with extruded pellet feed twice a day. A sexually mature female fish weighing 325 g and 23 cm in length and a sexually mature male fish weighing 298 g and 21 cm in length were used to collect eggs and semen, respectively.

### Transcriptional Knock-down of *mstn*

#### Sequence Design and Transfection Reagents Preparation

**Fig. 1:**
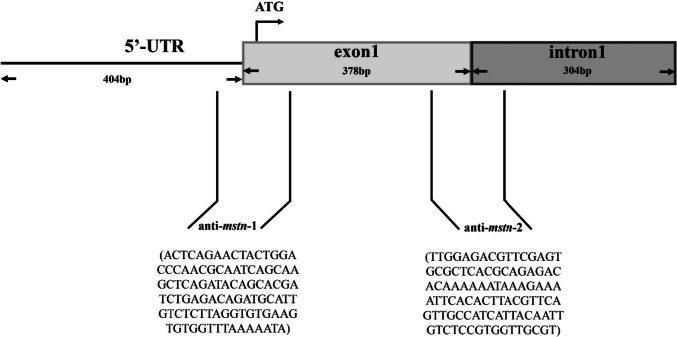
Design and action site of double-antisense RNA

The designed sequences (*mstn*: XM_003458832.5) (Fig. 1) were synthesized by the Jinweizhi Biotechnology Co., Ltd. (Suzhou, China). The sequences were as follows: antisense RNA sequence 1 of *mstn*-1 (anti-*mstn*-1): ACTCAGAACTACTGGACCCAACGCAATCAGCAAGCTCAGATACAGCACGATCTGAGACAGATGCATTGTCTCTTAGGTGTGAAGTGTGGTTTAAAAATA; antisense RNA sequence 2 of *mstn*-2 (anti-*mstn*-2): TTGGAGACGTTCGAGTGCGCTCACGCAGAGACACAAAAAATAAAGAAAATTCACACTTACGTTCAGTTGCCATCATTACAATTGTCTCCGTGGTTGCGT.

Anti-*mstn*-1 and anti-*mstn*-2 were cloned into the pcDNA3.1 expression vector containing the strong CMV promoter and used as the template for subsequent PCR amplification (polyAF1: GCTTAGGGTTAGGCGTTTTGC and polyAR1: TCCCCAATCCTCCCCCTTGCTG). The amplification procedures and methods were as described in our previous study (Yan et al. 2022). The PCR amplification product (above, mixed at a 1:1 ratio) (treatment group), ultra-pure water (control group), or blank expression vector amplification product (negative control; NC group) was mixed with sperm preservation fluid (4% w/v sucrose, 3% v/v glycerol, and 1% v/v dimethyl sulfoxide) and lipofectamine 2000 (Thermo Fisher Scientific, Waltham, MA, USA) at a ratio of 2:31:2, and then the mixture was allowed to equilibrate for 30 min before the transfection procedure.

#### Artificial Insemination and Management of Experimental Fish

A sexually mature female was selected, and its abdomen was gently squeezed to release mature eggs. The eggs were collected in stainless steel basins. Buffer (see Yan et al. (2022) for buffer constituents) was added to keep the micropyle open, and then 0.8 mL transfection reagent was added for about 500 eggs. The mixture was mixed slowly for 15 min. Semen was extracted from a sexually mature male fish with a disposable dropper and added to each stainless steel basin containing eggs. The mixture was gently agitated with a feather. The fertilized eggs were cultured in an incubator, and the newly hatched larvae were collected after 96 h and fed four times daily. One month later, fish were selected and placed in 12 indoor tanks (four tanks per group) at a stocking density of 30 fish/m^3^. Feeding and water quality management were as described in the “Acclimation of Fish Before Experiment” section. The fish were fed for 180 days.

#### Detection of Positive Transfection Rate

The positive transfection rate was determined as described by Qiang et al. (2022). Briefly, fish were selected from the treatment, control, and NC groups, and the muscle tissue was dissected. Genomic DNA was extracted using the FastPure Cell/Tissue DNA Isolation Mini Kit (Vazyme Biotech Co., Ltd, Nanjing, China). The amplification procedures and methods were as described in our previous study (Qiang et al. 2022).

### Sampling

Food was withheld from the fish for 1 day before sampling. Three well-developed fish were selected from each tank and subjected to deep anesthesia using MS-222 solution (200 mg/L). Various characteristics were measured, and then the fillets were removed. Specifically, each fillet was obtained by cutting along the line of the abdomen, the back edge of the gill cover, the dorsal fin, the end of the dorsal fin, and the end of the anal fin. The bone was removed, and then the fillet was weighed. The growth performance was evaluated based on the growth rate and the fillet yield, which were calculated as follows:

Growth rate (WGR) = [final body weight (g) − initial body weight (g)]/initial body weight (g).

Fillet yield (*Y*_F_) = [fillet mass (FW)/body weight (BW)] × 100%.

Three further fish were selected from each tank. The muscle tissue was removed and divided into seven portions. Each portion was placed in a cryovial and frozen with liquid nitrogen. The seven portions were used for nutrient composition determination, lipid index determination, fatty acid composition determination, RNA extraction, and western blot analysis.

Another three fish were selected from each tank, and their muscle tissue was divided into two parts: one was frozen for oil red O staining, and the other was fixed in 4% v/v paraformaldehyde for skeletal muscle fiber analysis.

### Determination of Meat Quality Indexes

#### Nutrient Composition

The moisture content was determined using the direct drying method at 105 °C, according to GB5009.3–2016. According to GB5009.4–2016, the crude ash in samples was determined by the gravimetric method after burning at 550 °C. The crude fat content was determined after Soxhlet extraction using an automatic crude fat analyzer (Qingdao Kechuang Quality Testing Co., Ltd, Qingdao, China) according to GB5009.6–2016. The crude protein content was determined using the Kjeldahl method with an automatic crude protein analyzer (Qingdao Kechuang Quality Testing Co) according to GB500.5–2016.

#### Lipid Index

The TG and total cholesterol (T-CHO) contents were determined using the A110-1–1 kit and the A111-1–1 kit, respectively (Jiancheng Bio Inc., Nanjing, China), according to the manufacturer’s instructions.

#### Fatty Acid Composition

Fatty acids were extracted using the method described previously (Bao et al. 2018). Each frozen sample was freeze-dried until no moisture remained. The freeze-dried samples were powdered with a mortar and pestle, placed in a plastic zipper storage bag, and sent to the Qingdao Kechuang Quality Co., Ltd, for the detection of fatty acid components.

### Muscle Tissue Section Preparation and Analysis

The method for preparing muscle sections was as described previously (Gao et al. 2019). Muscle tissue was fixed in 4% v/v paraformaldehyde for 4 days followed by paraffin sectioning and hematoxylin–eosin staining. The muscle tissue was flash-frozen and then sectioned with a freezing microtome (Microm International GmbH, Walldorf, Germany). The muscle tissue was examined under a microscope (Eclipse Ci-L, Nikon, Tokyo, Japan) and photographed. Individual muscle fibers were outlined; the cross-sectional diameter was determined with Image-Pro Plus 6.0 (Media Cybernetics, Silver Spring, MD, USA), and the number of muscle fibers in each visual field was counted. To visualize lipid droplets in the muscle tissue, we stained the frozen sections with oil red O, as described previously (Amali et al. 2006). The tissue sections were washed with phosphate-buffered saline, fixed for 24 h, stained with oil red O working solution for 30 min, and then observed under the microscope. At least six images per treatment group were analyzed using Image-Pro plus 6.0 to calculate the area of lipid droplets.

### Western Blot Analysis

Polyclonal antibodies against myostatin protein and *β*-actin protein were obtained by immunizing New Zealand white rabbits. After the rabbit serum titer was identified using an enzyme-linked immunoassay kit (Jiancheng Bio Inc., Nanjing, China), the best rabbit serum for each antigen was purified by affinity chromatography column chromatography, and then the protein was purified by sodium dodecyl sulfate–polyacrylamide gel electrophoresis (SDS-PAGE). The antibodies were synthesized by Hua’an Biotechnology Co., Ltd. (Hangzhou, China), and ELISA kits were used to detect antibodies in New Zealand white rabbit serum.

For western blot analysis, 50-mg muscle tissue blocks were cut, ground with liquid nitrogen, and mixed with 500 µL RIPA cracking solution. The mixture was incubated on ice for 30 min and then centrifuged at 4 °C at 12,000 r/min for 15 min. The supernatant was used for further analyses. The protein concentration in the supernatant was determined using a BCA protein assay kit (Sigma-Aldrich Inc., St Louis, MO, USA). According to the results of BCA, the solution was diluted to the appropriate final concentration, and SDS-PAGE protein loading buffer (5 ×) was added. The sample was heated at 100 °C for 10 min, separated by SDS-PAGE, and then the separated proteins were transferred to a PVDF membrane by wet rotation. The membrane was blocked with blocking solution (TBST solution with 5% w/v skim milk powder) at room temperature for 1 h. The primary antibody was diluted with diluted with the blocking solution and incubated with the blocked PVDF membrane overnight at 4 °C. The membrane was washed with PBST and then incubated with the secondary antibody diluted in blocking solution at 1:5000 at room temperature for 1 h. The color was detected using Immobilon Western HRP (ECL) (Merck KgaA, Darmstadt, Germany). Β-actin was used as the internal reference protein.

### RNA Extraction and cDNA Synthesis

We extracted RNA from muscle tissue using the Trizol method as described elsewhere (Yan et al. 2022). The integrity of the extracted RNA was checked by electrophoresis. The total extracted RNA was stored at − 80 °C. Using the extracted RNA as a template, cDNA was prepared using HiScript III RT SuperMix for qPCR (+ gDNA wiper) (Vazyme), according to the manufacturer’s instructions, and then stored at − 20 °C.

### Real-time Fluorescent Quantitative PCR

Real-time fluorescent quantitative PCR (qRT-PCR) was performed using SYBR® Premix Ex Taq™ (TaKaRa, Dalian, China) according to the manufacturer’s instructions, with gene-specific primers (Table 1). The qRT-PCR procedure was the same as described previously (Yan et al. 2022). Each reaction was replicated three times.

**Table 1:** Sequences of primers used for qRT-PCR

**Gene name**	**Gene product name**	**Primer sequence (5′–3′)**
*mstn*	Myostatin	F: TGCGTTGGGTCCAGTAGTTC
*lpl* *eci1* *acaa2* *foxo3* *lepr* *acadvl* *myod1* *myog* *myf5*	Lipoprotein lipase Enoyl-CoA delta isomerase 1 Acetyl-CoA acyltransferase 2 Forkhead box o3 Leptin receptor Acyl-CoA dehydrogenase very long chain Myogenic differentiation 1 Myogenin Myogenic factor 5	R: CCTTCATTCGCAGCTTGCTC F: CACTGAATGGCTCACCGACT R: GTTACCGTCCAGCCGTGTAT F: ACATCGGACCACAGAGATGG R: CTGTCTGGCGTGATCTGGTA F: GCTGATGCACCCTACATTGC R: AGGACCACCTCTGAATCCCT F: CTCGCACAAACTCCAATGCC R: CACAGCCTTCCCATTCGTCT F: CGGCTCGTTTTGTGAGAGGT R: GAATCTCAGTCGCTGCCGAA F: CCCATGAAGGACCCCAAGAC R: CTTGAATCCGCCTCCGAGTT F: CCGCTGATGATTTCTATG R: GATGAGGATGAAGAGGAG F: CCACAATGGAGGTCAAGG R: AGAGTGTCGTCGTCAAGC F: CGTGAGGCACATAATAAATAAC R: TGATGCTGAAAGCGACTG
*β-actin*		F: CCACACAGTGCCCATCTACGA
		R: CCACGCTCTGTCAGGATCTTCA

### RNA-Sequencing

The RNA extracted from muscle tissue of fish in the treatment and NC group as described in the “RNA Extraction and cDNA Synthesis” section was sequenced. We constructed four NC (NC_1, NC_2, NC_3, NC_4) and four treatment (MSTN_1, MSTN_2, MSTN_3, MSTN_4) sequence libraries. Paired-end sequencing was performed using an Illumina Novaseq™ 6000 instrument (Illumina, San Diego, CA, USA). We used Cutadapt software to delink the raw data acquired in FastQ and to remove low-quality and repetitive sequences. We obtained bam files by comparing the clean data with the Nile tilapia genome using HISAT2 software. Initial assembly of genes or transcripts was performed using StringTie software, and the initial assembly results of all samples were combined. Differentially expressed genes (DEGs) between the treatment and NC group were detected with DESeq2 (Jiang et al. 2020), according to the criteria |log2 fold change|≥ 1 and *p* < 0.05 (Li et al. 2021). The DEGs were subjected to GO and KEGG analyses. We selected 10 DEGs to verify the RNA-sequencing results by qRT-PCR as described in the “Real-time Fluorescent Quantitative PCR” section.

### Statistical Analysis

Statistical analyses were conducted using IBM SPSS Statistics v 22.0 (SPSS Inc., Chicago, IL, USA). Data shown in figures and tables are the mean ± standard error (mean ± SE). We used Shapiro–Wilk’s and Levene’s tests to test the normality and homogeneity of variance of the data and one-way analysis of variance (ANOVA) to compare mean values among treatments. Differences were considered significant at *p* < 0.05. Multiple comparisons were conducted using Tukey’s method.

## Results

### Determination of Positive Transfection Rate

**Fig. 2:**
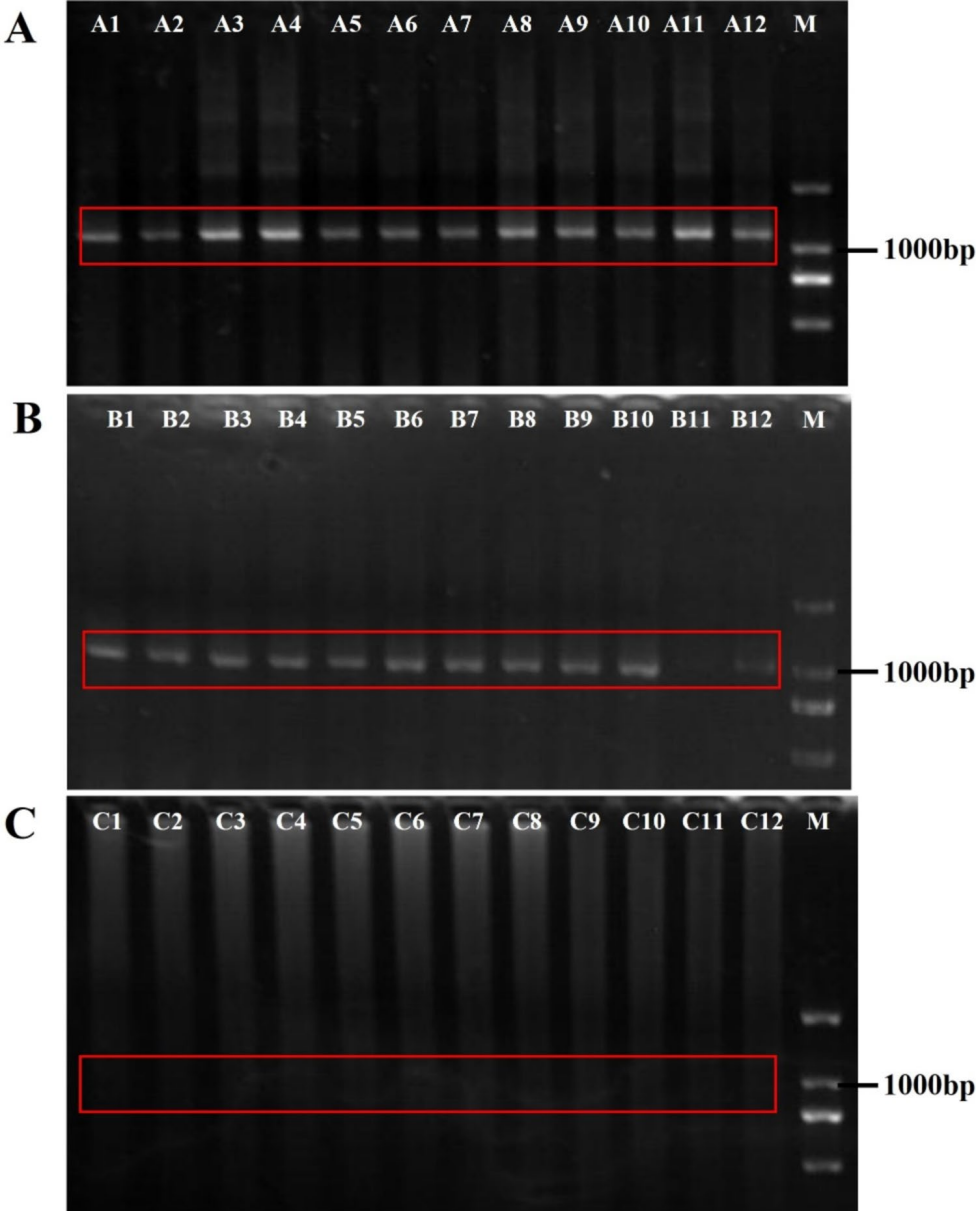
Detection of positive transfection rate by analysis of muscle tissue. **A** PCR analysis of muscle tissue from the treatment group transfected with antisense RNA fragment, with an obvious band at about 1100 bp (plasmid 1000 bp + antisense RNA fragment of about 100 bp). A1–A12 (red box) represent 12 replicates from the treatment group; **B** PCR analysis of muscle tissue from the negative control (NC) group with a band of 1000 bp (plasmid 1000 bp). B1–B12 (red box) represent 12 replicates; **C** PCR analysis of muscle tissue from the control group, with no obvious band at the position of 1000–1100 bp. C1–C12 represent 12 replicates

The muscle tissue was analyzed to detect the positive transfection rate. The results showed that the treatment group and the NC group had a specific band at around 1100 bp and 1000 bp, respectively, whereas the control group had no band (Fig. 2). The sequencing work was done by Genewiz Biotechnology Co., Ltd., Suzhou, China, and the results confirmed that the antisense RNA sequences were present in the fish in the treatment group, the plasmid sequence was present in the fish in the NC group, and no extra plasmid or antisense sequence was present in fish in the control group.

### *mstn* Knock-down Decreased *mstn* Transcript and Myostatin Protein Levels in Muscle Tissue

**Fig. 3:**
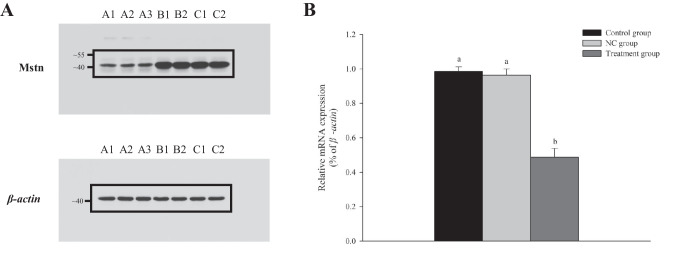
*mstn* transcript and myostatin protein levels in the control group, NC group, and treatment group. **A** Western blot analyses showing relative myostatin levels in muscle tissue in treatment and control groups: A1–A3, treatment group; B1–B2, control group; C1–C2, negative control (NC) group. *β-actin* was the internal reference in each group; **B** transcript levels (mean ± SE, *n* = 12 replicates per group) of *mstn* in muscle tissue of treatment, control, and NC groups as determined by qRT-PCR. Different lowercase letters indicate significant differences (*p* < 0.05)

The myostatin protein was detected in the Nile tilapia muscle tissue (Fig. 3A). The results of qRT-PCR and western blot analyses showed that *mstn* transcript and myostatin protein levels were significantly lower in the treatment group than in the NC and control groups (*p* < 0.05) (Fig. 3B).

### *mstn* Knock-down Enhanced Body Growth and Led to Muscle Fiber Hyperplasia

**Fig. 4:**
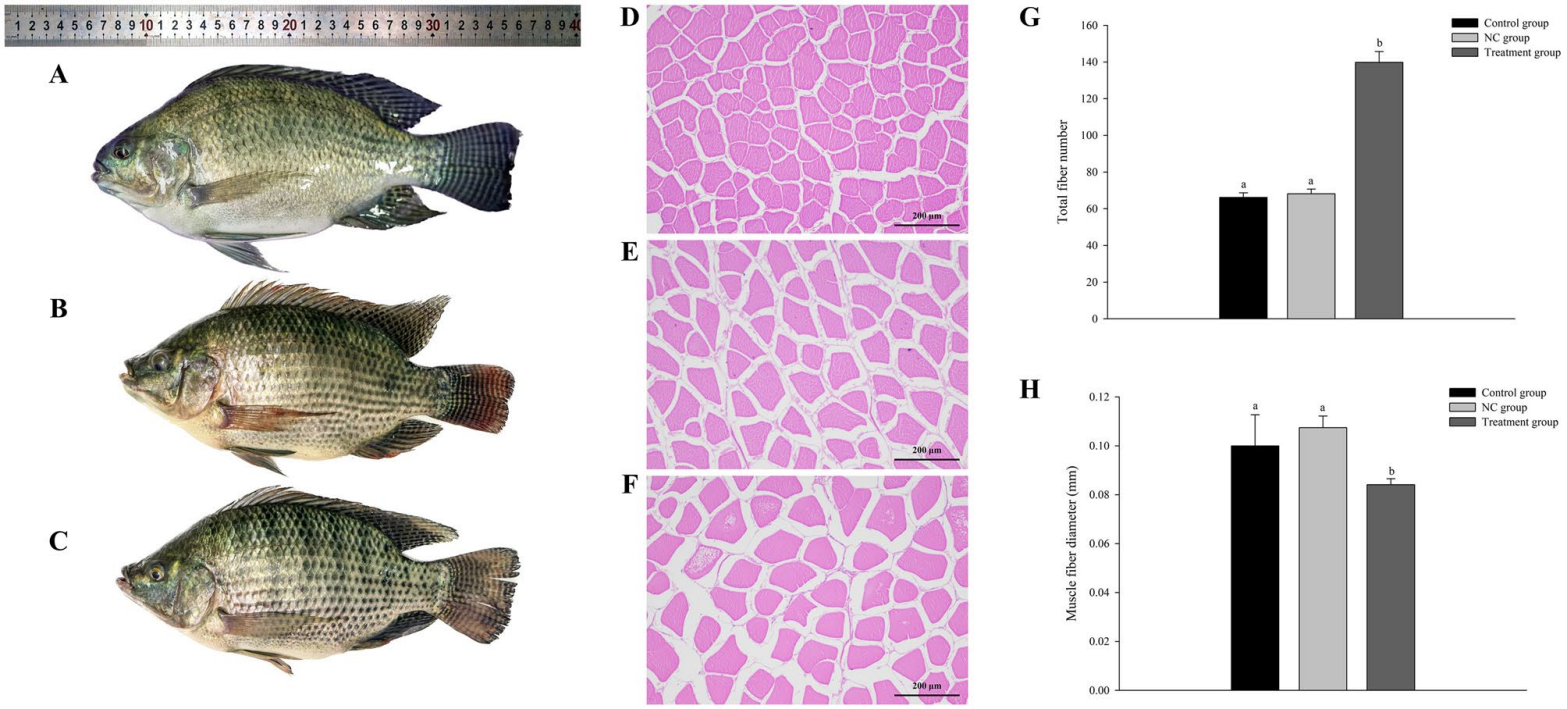
Appearance and muscle characteristics of Nile tilapia in control, NC, and treatment groups. **A** Figure shows appearance of fish in the treatment group, **B** NC group, and **C** control group. **D** Hematoxylin–eosin staining of paraffin sections of muscle tissue in the treatment group, **E** NC group, and **F** control group. **G** Total number of fibers in each group. **H** Muscle fiber diameter in each group. Different lowercase letters indicate significant differences (*p* < 0.05)

We measured morphological indicators (body weight, body thickness, body length, body height, and fillet yield) of the fish in the three groups (Table 2). Compared with fish in the NC and control groups, those in the treatment group were larger with significantly increased body weight and body length (*p* < 0.05, Fig. 4A–C). Generally, the WGR and fillet yield ratios were higher in the treatment group than in the NC and control group (*p* < 0.05) (Table 2). Analyses of hematoxylin–eosin–stained sections indicated that the treated group exhibited a higher degree of muscle fiber density compared to the NC and control groups (Fig. 4D–F). Additionally, the number of muscle fibers was markedly greater in the treatment group than in both the NC and control groups (Fig. 4G) However, the muscle fiber diameter in the treatment group was significantly smaller compared to both the control group and the NC group (Fig. 4H).

**Table 2:** Growth performance indexes in control, NC, and treatment groups

**Measurement**	**Control group (*n* = 12)**	**Negative control (NC) group (*n* = 12)**	**Treatment group (*n* = 12)**
Initial body weight (g) Final body weight (g) Initial body thickness (cm) Final body thickness (cm) Initial body length (cm) Final body length (cm) Initial body height (cm) Final body height (cm) Height/length Thickness/length	39.24 ± 1.43 229.37^b^ ± 10.34 18.02 ± 0.44 3.24 ± 0.63 10.19 ± 0.09 17.76^b^ ± 1.43 4.01 ± 0.06 7.35^b^ ± 0.63 0.41 ± 0.05 0.18 ± 0.02	38.69 ± 1.72 231.42^b^ ± 12.13 18.27 ± 0.25 3.33 ± 0.52 10.06 ± 0.18 18.23^b^ ± 1.24 4.03 ± 0.12 7.42^b^ ± 0.78 0.41 ± 0.07 0.18 ± 0.01	37.81 ± 0.80 296.36^a^ ± 14.23 18.15 ± 0.38 3.74 ± 0.49 10.25 ± 0.15 20.17^a^ ± 3.21 4.05 ± 0.18 8.21^a^ ± 0.51 0.41 ± 0.02 0.19 ± 0.03
Growth rate (WGR) Specific growth rate (SGR)	4.85^b^ ± 1.25 0.01 ± 0.15	4.98^b^ ± 0.98 0.01 ± 0.14	6.84^a^ ± 1.32 0.01 ± 0.2

Data were analyzed by one-way ANOVA. Differences among three groups were detected using Tukey’s multiple comparisons test (*p* < 0.05). Different lowercase letters indicate significant differences among groups (*p* < 0.05)

### *mstn* Knock-down Affected Meat Quality

**Fig. 5:**
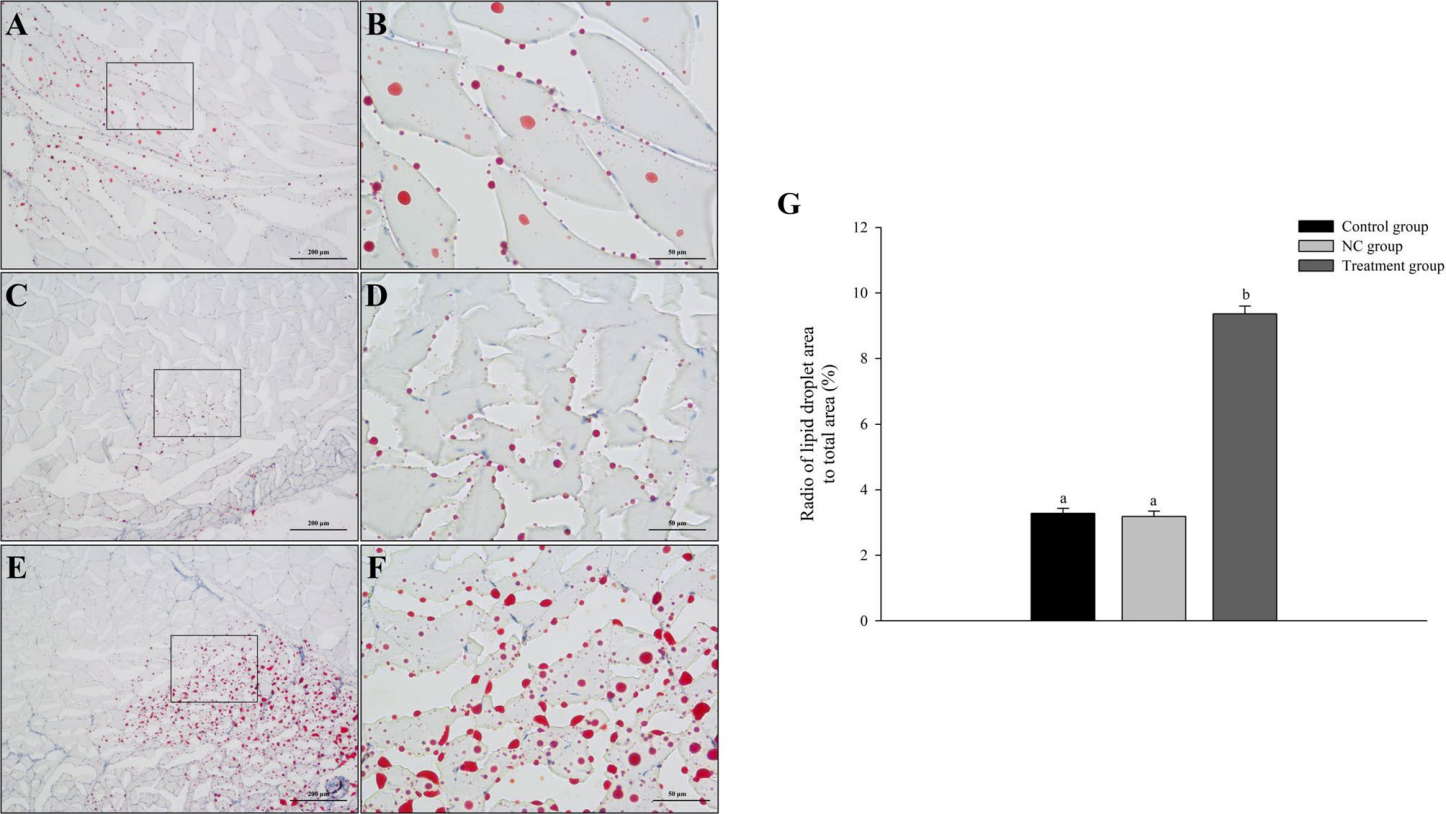
Representative sections of muscle tissue from Nile tilapia in (*n* = 12 replicates per group): **A** control group at 100 × and **B** 400 ×, **C** NC group at 100 × and **D** 400 ×, **E** treatment group at 100 × and **F** 400 ×. **G** Radio of lipid droplet area to total area in each group at 400 ×. Different lowercase letters indicate significant differences (*p* < 0.05)

We performed oil red O staining on skeletal muscle tissue to study the effect of *mstn* knock-down on the muscle quality of Nile tilapia. Compared with the NC and control groups, the treatment group showed increased fat accumulation in muscle tissue (Fig. 5). Analyses of nutrient composition and the lipid index revealed drastic increases in fat and TG in muscle tissue of fish in the treatment group compared with those in the control and NC groups (*p* < 0.05). The muscle tissue of Nile tilapia contained 21 fatty acids, including seven saturated fatty acids (SFA), five monounsaturated fatty acids (MUFA), and nine polyunsaturated fatty acids (PUFA). Compared with the control group and the NC group, the treatment group showed dramatically increased contents of SFA, PUFA, MUFA, and n-6 PUFA, and n-3 PUFA (*p* < 0.05) (Table 3).

**Table 3:** Muscle nutrient composition, lipid index, and fatty acid composition

**Measurement**	**Control group (*n* = 12)**	**Negative control (NC) group (*n* = 12)**	**Treatment group (***n*** = 12)**
Moisture (g/100 g)	76.41 ± 2.52	77.35 ± 1.85	74.50 ± 1.98
Crude ash (g/100 g)	1.27 ± 0.24	1.28 ± 0.15	1.26 ± 0.23
Crude fat (g/100 g)	2.76^a^ ± 0.54	2.83^a^ ± 0.45	4.00^b^ ± 0.31
Crude protein (g/100 g)	19.30 ± 1.43	18.87 ± 1.32	18.84 ± 1.29
TG content (mmol/g muscle tissue)	4.32^a^ ± 1.21	4.43^a^ ± 1.05	6.26^b^ ± 0.98
T-CHO content (mmol/g muscle tissue)	1.54 ± 0.33	1.46 ± 0.34	1.44 ± 0.21
SFA content (g/100 g)	0.67^a^ ± 0.22	0.65^a^ ± 0.32	1.25^b^ ± 0.21
PUFA content (g/100 g)	0.35^a^ ± 0.04	0.34^a^ ± 0.13	0.98^b^ ± 0.11
MUFA content (g/100 g)	0.53^a^ ± 0.13	0.56^a^ ± 0.14	1.35^b^ ± 0.27
n-6 PUFA content (g/100 g)	0.26^a^ ± 0.11	0.31^a^ ± 0.15	0.73^b^ ± 0.18
n-3 PUFA content (g/100 g)	0.07^a^ ± 0.02	0.05^a^ ± 0.03	0.18^b^ ± 0.05

Data were analyzed by one-way ANOVA. Differences among the three groups were detected using Tukey’s multiple comparisons test (*p* < 0.05). In each row, different lowercase letters indicate significant differences among groups (*p* < 0.05)

### Effect of *mstn* Knock-down on Muscle Development and Lipid Metabolism

**Fig. 6:**
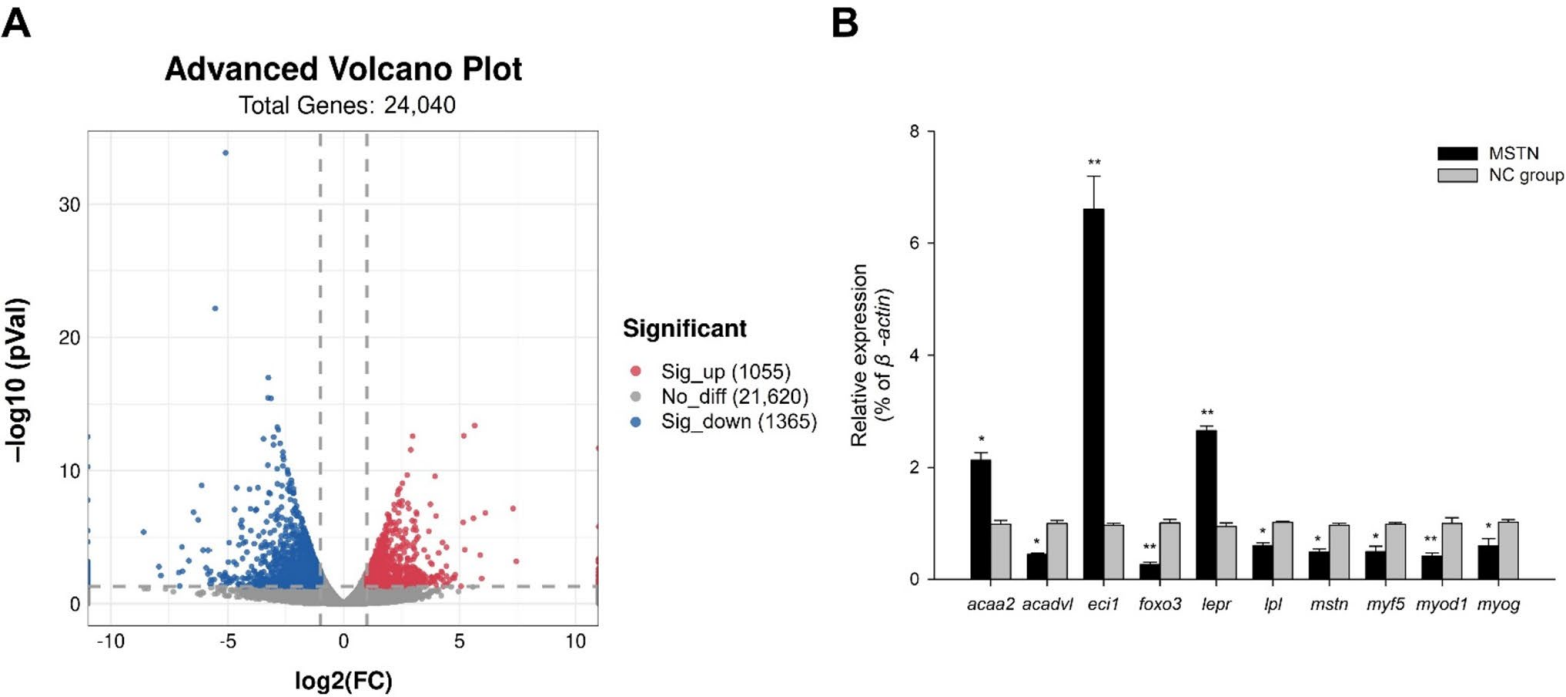
**A** Volcano plot of differentially expressed genes (DEGs) in Nile tilapia with knocked-down *mstn* vs. NC. Dots: blue, down-regulated DEGs in treatment vs. NC; red, up-regulated DEGs; gray, genes with no significant difference in expression; **B** transcript levels of DEGs based on qRT-PCR analyses (*n* = 9 replicates per group). Ten DEGs were selected for qRT-PCR verification. Asterisk (*) indicates significant difference (*p* < 0.05) between the treatment group and NC group

After the transcriptomes of the muscle tissue were sequenced and the raw reads were processed, the number of clean reads ranged from 46,455,136 to 52,565,182. The Q20 values ranged from 97.44 to 98.30%, and the Q30 values ranged from 93.54 to 95.43% (Table 4). In total, 29,528 genes (14,731 up-regulated and 14,797 down-regulated genes in the treatment group vs. the NC group) were sequenced in this experiment. A total of 2420 significant DEGs in the *mstn*-knock-down group vs. the NC group (1055 up-regulated and 1365 down-regulated genes) met the screening criteria (Fig. 6A).

**Table 4:** Overview of RNA-sequencing data and quality filtering

**Sample**	**Raw reads**	**Valid reads**	**Valid bases (***G***)**	**Valid ratio (reads)**	**Q20 (%)**	**Q30 (%)**
MSTN_1	56,366,592	51,526,252	7.73	91.41	98.16	95.09
MSTN_2	53,536,100	48,951,576	7.34	91.43	98.28	95.32
MSTN_3	48,419,310	44,191,280	6.63	91.26	98.19	95.15
MSTN_4	56,300,514	51,669,906	7.75	91.77	98.03	94.84
NC_1	50,135,852	46,455,136	6.97	92.65	98.30	95.37
NC_2	57,182,986	52,565,182	7.88	91.92	98.28	95.35
NC_3	56,482,390	51,992,736	7.80	92.05	98.30	95.43
NC_4	57,457,366	51,315,170	7.70	89.3	97.44	93.54

We selected 10 DEGs for validation by qRT-PCR analysis. The experimental results were consistent with those obtained from the RNA-sequencing data (coefficient of determination, *R*^2^ > 0.9), thus confirming the accuracy of the sequencing results. The transcript levels of *mstn*, *myod1* (encoding myogenic differentiation 1), *lpl*, *foxo3* (encoding forkhead box o3), *myog* (encoding myogenin), *acadvl* (encoding acyl-coa dehydrogenase very long chain), and *myf5* (encoding myogenic factor 5) were dramatically down-regulated in the treatment group compared with the NC group. (*p* < 0.05). The transcript levels of *eci1*, *acaa2* (acetyl-CoA acyltransferase 2), and *lepr* (encoding leptin receptor) were dramatically up-regulated in the treatment group compared with the NC group (*p* < 0.05) (Fig. 6B).

#### Functional Annotation of DEGs by GO and KEGG Analysis

**Fig. 7:**
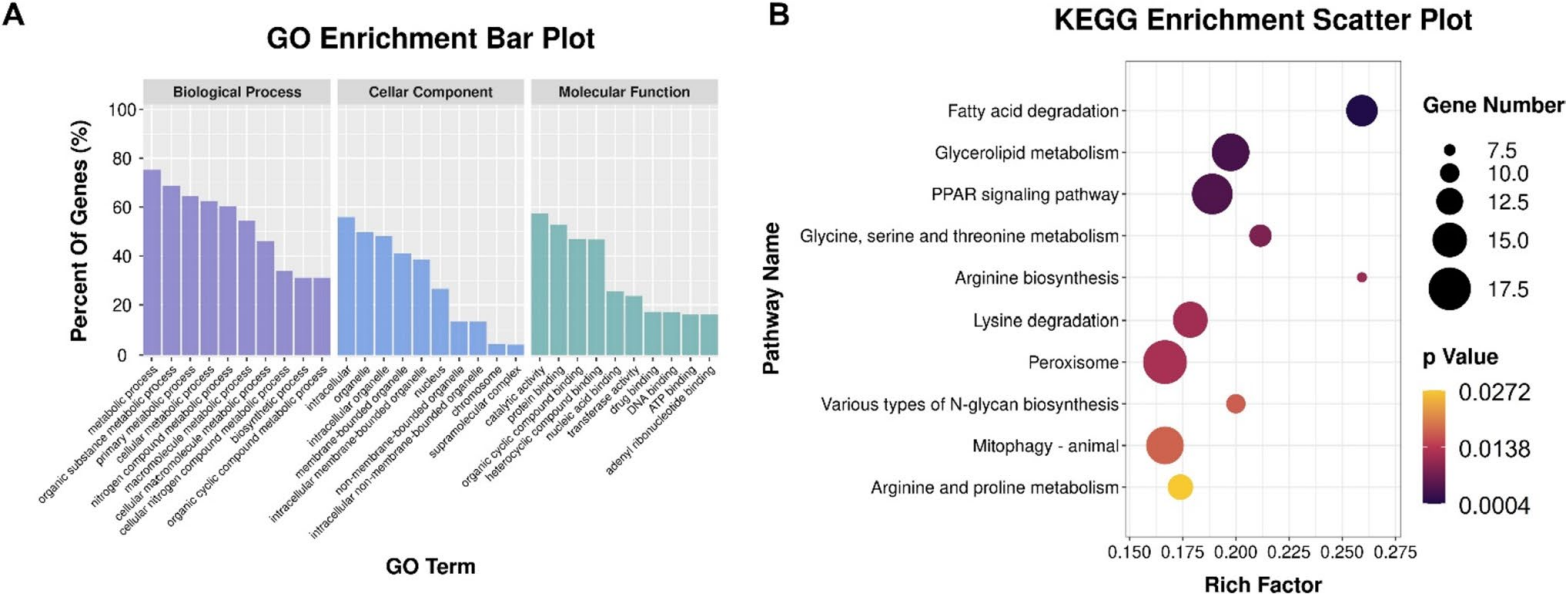
**A** Gene Ontology (GO) categories enriched with DEGs in muscle tissue of Nile tilapia with knocked-down *mstn*. Each annotated sequence was assigned at least one GO term in the following categories: biological process, cellular component, or molecular function; **(B)** Kyoto Encyclopedia of Genes and Genomes (KEGG) pathways associated with lipid metabolism that were enriched with DEGs in Nile tilapia with knocked-down *mstn* (treatment vs. NC)

The DEGs were functionally annotated by GO enrichment and KEGG pathway analyses. The DEGs were dramatically enriched in 341 GO terms, including 227 in the biological process category (metabolic process, organic substance metabolic process, and primary metabolic process), 37 in the cellular component category (intracellular, organelle, and intracellular organelle), and 77 in the molecular function category (catalytic activity, protein binding, and organic cyclic compound binding) (Fig. 7A). In the KEGG analysis, the DEGs were dramatically enriched in 10 signaling pathways: fatty acid degradation; glycerolipid metabolism; peroxisome proliferator activated receptors (PPAR) signaling pathway; glycine, serine, and threonine metabolism arginine biosynthesis; lysine degradation; peroxisome; various types of N-glycan biosynthesis, mitophagy – animal; and arginine and proline metabolism (Fig. 7B).

#### Signaling Pathways Related to Lipid Metabolism and the Endocrine System

**Fig. 8:**
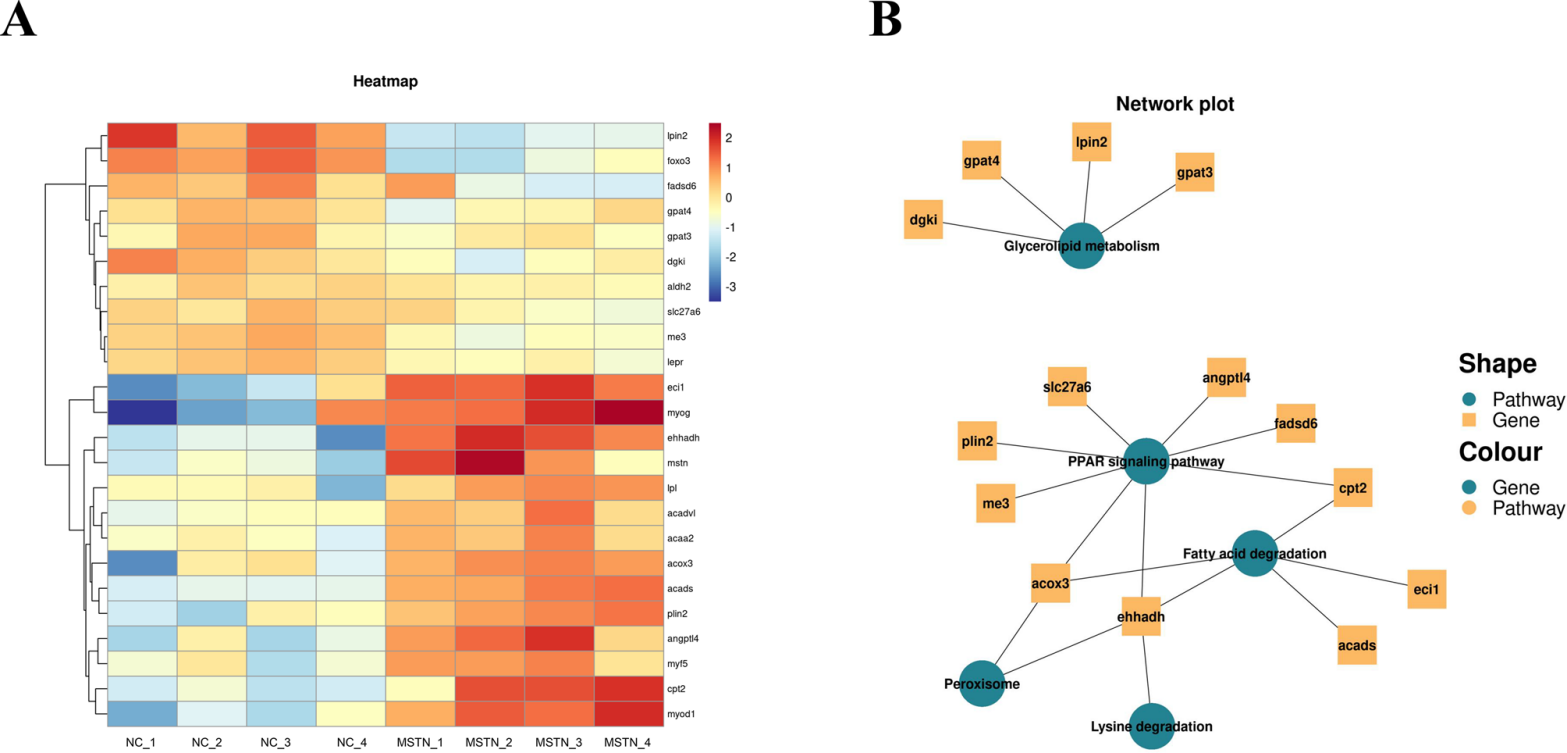
**A** Abnormal lipid metabolism caused by *mstn* knock-down in Nile tilapia. Heat map of 24 DEGs identified in KEGG pathways associated with lipid metabolism (criteria for DEGs: |log2 fold change|≥ 1 and *p* < 0.05). Colors are scaled per row. Colors indicate level of gene expression from highest (red) to lowest (blue); **B** enrichment analysis of lipid metabolism-related genes and pathways

The DEGs related to lipid metabolism were enriched in fatty acid degradation, glycerolipid metabolism, and PPAR signaling pathways. The KEGG analysis revealed that 18 major DEGs were involved in the above three signaling pathways. The significantly up-regulated DEGs (*p* < 0.05) were *eci1* (encoding enoyl-coa delta isomerase 1), *lpin2* (encoding phosphatidate phosphatase LPIN2), *angptl4* (encoding angiopoietin-related protein 4), *cpt2* (encoding carnitine O-palmitoyltransferase 2), *dgki* (encoding diacylglycerol kinase iota isoform X1), and *gpat3* (encoding 1-acyl-sn-glycerol-3-phosphate acyltransferase gamma). The significantly down-regulated DEGs (*p* < 0.05) were *ehhadh* (encoding peroxisomal bifunctional enzyme), *acads* (encoding short-chain specific acyl-CoA dehydrogenase), *me3* (encoding NADP-dependent malic enzyme), *plin2* (encoding perilipin-2), *gpat4* (encoding 1-acyl-sn-glycerol-3-phosphate acyltransferase delta isoform X1), and *lpl* (encoding lipoprotein lipase) (Fig. 8).

## Discussion

The down-regulation of *mstn* results in compensatory muscle growth, and this provides a basis for developing strategies to promote fish growth. To date, few studies have focused on *mstn* in Nile tilapia. We introduced antisense RNA into eggs of Nile tilapia to knock-down *mstn* gene expression. In future studies, we will further optimize the promoters and plasmids to ensure stable inheritance and expression of antisense RNA in offspring. In addition, we will further explore how the transfection sequence enters the egg through the micropyle (Cao et al. 2022). The establishment of a hereditarily stable knocked-down *mstn* Nile tilapia line offers a good model for investigating the functional role of *mstn*.

### Knock-down of *mstn* Promotes Growth and Muscle Development

The muscle mass directly affects the edible portion of fish, which is critical for aquaculture production (Ohama et al. 2020). Previous studies have shown that *mstn* mutants display increased muscle mass and a double-muscle phenotype, not only in mammals (Kambadur et al. 1997; Lee 2007; McPherron et al. 1997; Mosher et al. 2007) but also in fish (Chisada et al. 2011; Gao et al. 2019). At the age of 180 days, Nile tilapia with knocked-down *mstn* showed increased body weight, body thickness, body height, and fillet yield compared with those in the control group. The muscle mass is determined by the number and size of muscle fibers, and the number of muscle fibers is also an important factor in determining fish size (Biga and Goetz 2006). The growth analysis results showed that the number of muscle fibers was increased but the diameter of muscle fibers did not change significantly upon *mstn* knock-down. Therefore, we concluded that the increase in body size and meat yield of fish in the treatment group was caused by the increase in the number of muscle fibers. The null mutation of myostatin protein (MSTN) induced muscle fiber hyperplasia, but not hypertrophy, to increase the muscle mass by 150% in male tilapia (Wu et al. 2023). However, incomplete blocking of endogenous myostatin protein led to hyperplasia or hypertrophy. A missense mutation of *mstn* caused hyperplasia but not hypertrophy in mice muscle tissue, whereas a dominant negative *mstn* genotype resulted in muscle hypertrophy but not hyperplasia (Acosta et al. 2005). Our results show that only the number of muscle fibers was dramatically increased in the treatment group, suggesting that *mstn* knock-down resulted in muscle tissue hyperplasia rather than hypertrophy in Nile tilapia, and that the main reason for weight gain was muscle fiber hyperplasia. The effect of *mstn* on muscle growth varies among fish species (Zhang et al. 2020). For example, *mstn* genome-edited catfish (*Ictalurus punctatus*) displayed muscle fiber hyperplasia (Khalil et al. 2017). Yellow catfish (*Pelteobagrus fulvidraco*) with knocked-out *mstna* exhibited an increased number of myofibers, but their size was decreased (Zhang et al. 2020). Knockout of *mstn* in medaka (*Oryzias latipes*) caused muscle hypertrophy but not hyperplasia (Yeh et al. 2017). However, *mstn* genome-edited common carp (*Cyprinus carpio*) exhibited an increased number of muscle fibers and hypertrophy (Zhong et al. 2016). The hyperplasia of muscle fibers without hypertrophy observed in this study may be due to incomplete inhibition of *mstn* expression.

### Knock-down of *mstn* Affects Adipogenesis in Muscle Tissue

Myostatin is not only a key factor in muscle development, but also an important regulatory factor in lipid metabolism. Adipose tissue functions to regulate energy metabolism (Madureira et al. 2018). Lipids are signaling molecules and energy sources for growth and reproduction, and the amount of lipid produced in muscles can regulate the muscle mass of fish (Johnson 2009). The *mstn* gene is mainly expressed in adult mammalian skeletal muscle and affects not only muscle development but also adipogenesis (Rodgers and Garikipati 2008). However, the relationship between *mstn* and adipogenesis is controversial—*mstn* may have dual regulatory effects on adipogenesis. The metabolic mechanism of muscle fat production varies among different animals. Artaza et al. (2005) found that *mstn* could promote the transformation of mesenchymal cells into adipocytes. Lin et al. (2002) constructed a knockout model of *mstn* in mice and found that they displayed reduced adipogenesis, accompanied by decreased leptin content in the body (Pantoja et al. 2008). In contrast, Gao et al. (2019) found that *mstnb*-deficient zebrafish (*Brachydanio rerio var*) formed less subcutaneous and visceral adipose tissue and accumulated more fat in muscle tissue. The opposite results in mammals and fish may be a consequence of excess lipid tending to transfer from fat-prone sites to skeletal muscle in fish with down-regulated *mstn* (Gao et al. 2019). We obtained similar results, in that down-regulation of *mstn* resulted in increased adipogenesis in muscle tissue and the significant increase in TG led to changes in fatty acid content composition. Intramuscular fat is a site of TG storage and is rich in phospholipids and TC, all of which affect the flavor and quality of the meat. To a certain extent, higher IMF content is associated with better flavor and texture of fish meat. Fish meat is rich in highly unsaturated fatty acids that are beneficial for human health. The fatty acid composition in fish tissues also reflects the protective effect of fatty acids against oxidative damage. In this study, the treatment group showed increased contents of SFA, PUFA, MUFA, n-6 PUFA, and n-3 PUFA, indicating that *mstn* affects the fatty acid profile in muscle tissue. In addition, the treatment group showed increased MUFA content, indicating that *mstn* knock-down inhibited fatty acid oxidation. Thus, Nile tilapia with knocked-down *mstn* not only showed a higher level of muscle development, but also increased adipogenesis in muscle tissue, both of which are important factors in achieving a high yield of farmed fish. The specific mechanism underlying this phenomenon needs to be further investigated.

### Molecular Mechanism by Which *mstn* Affects Muscle Development and Lipid Metabolism

The inhibition of skeletal muscle development by *mstn* can be explained by its effects on the proliferation and differentiation of myoblasts (Yang et al. 2007) and protein synthesis (Welle et al. 2006), as well as signal transduction of specific genes and pathways (Ayuso et al. 2015; Wu et al. 2018). Fatty acid degradation (Grabner et al. 2021) and peroxisomes (Okumoto et al. 2020) are involved in the metabolism of lipid and energy during growth. Glycerolipid metabolism may regulate skeletal muscle function and the IMF level (Xu et al. 2021). The PPAR signaling pathway regulates development and metabolism and is associated with adipogenesis in the muscle (Manickam et al. 2020). Previous studies have also shown that glycine, serine and threonine metabolism (Cheng et al. 2019), arginine biosynthesis (Chen et al. 2018), lysine degradation (Goda et al. 2021; Jin et al. 2019), and arginine and proline metabolism (Wu et al. 2009) regulate the degradation and synthesis of skeletal muscle proteins and participate in the induction of skeletal muscle growth and lipid metabolism. A similar phenomenon was found in this experiment. Fatty acid degradation and the PPAR signaling pathway jointly control muscle development. The results of this experiment showed that the expressions of *acox3*, *ehhadh*, *eci1*, *acaa2*, and *cpt2* belonging to both pathways were significantly changed.

Among the core genes that regulate muscle development, *mstn* is the most important negative regulator (Parsons et al. 2006). Generally, *mstn* keeps muscle cells in G0/G1 and G2 phases by regulating the binding of Myog and Smad proteins, thereby restricting the differentiation and proliferation of myoblasts (Li et al. 2014). Previous studies have shown that the regulation of *mstn* is a very complex process. The Myod gene family controls the proliferation and differentiation of muscle cells and is closely related to the number and size of muscle fibers, so it plays a very important role in meat quality and flavor. Members of the Myod gene family such as *myod*, *myog*, and *myf5*, encode different transcription factors that individually or synergistically control key regulatory factors in skeletal muscle generation. Foxo4, myod1, and *myog* regulate myogenesis, acting successively in the signaling chain (de la Serna et al. 2005; Zhao et al. 2011). When the activity of myostatin is inhibited, hyperplasia and hypertrophy may result from an imbalance between the proliferation and differentiation of myoblasts. In mammals, *myf5* and *myod* activate stem cells of myoblasts and induce their differentiation into myoblasts (Kablar et al. 1997), thereby playing an important role in the proliferation of muscle fibers (Oldham et al. 2001). Myog is important at the later stage of muscle differentiation, especially the late stage of differentiation during embryonic myogenesis and hypertrophy during postnatal myogenesis (Venuti et al. 1995). In this study, *mstn* knock-down led to the down-regulation of *myod* and *myf5*, which resulted in unbalanced proliferation and differentiation of myoblasts. In turn, this resulted in a series of changes in muscle fibers. The down-regulation of *myog* may be the main reason for the hyperplasia of muscle fibers without hypertrophy. Although the expression of myogenic genes was down-regulated in the treatment group, fish in the treatment group still displayed myofiber hyperplasia. Thus, we conclude that *mstn* is the main gene involved in myofiber differentiation, and this led to down-regulation of myogenic gene expression because the expression of these genes is correlated. Some myogenic genes are also related to lipid metabolism due to the diversity of gene functions. We conclude that the pathway involving *myod* and other members of its family may not be the only pathway for the regulation of muscle gene expression and that there may be other alternative pathways.

In individuals with skeletal muscle fiber hyperplasia induced by *mstn* knock-down, the fat content was significantly increased. Determining why the IMF content increases upon *mstn* knock-down will clarify the molecular mechanism of metabolic regulation, and this information will be useful for producing new lines in the future using molecular technology. Genetics is an important factor in the regulation of IMF. Studies on the regulatory mechanism IMF are of great significance for artificially controlling adipogenesis. *Lepr* inhibits insulin secretion and increases energy intake in animals (Festuccia et al. 2006). In the treatment group in this study, *lepr* was significantly up-regulated, which further affected the lipid metabolism and energy use of fish muscle tissue. This was specifically manifested as increased adipogenesis in muscle tissue and an increased number of muscle fibers. The main function of the product of *lpl* is to hydrolyze TG into fatty acids and glycerol (Kershaw et al. 2007). Knock-down of *mstn* led to the down-regulation of *lpl*, resulting in increased TG levels. Our results show that knock-down of *mstn* leads to abnormal lipid metabolism in fish muscle tissue. Changes in the expression of genes related to lipid metabolism lead to increased energy intake, which promotes body growth and adipose production.

## Conclusions

Our results show that antisense RNA technology can inhibit the *mstn* transcript level and myostatin protein level in Nile tilapia and effectively promote weight gain. Abnormal lipid metabolism is the main reason for the changes in muscle quality in Nile tilapia.

### Supplementary Information

Below is the link to the electronic supplementary material.

Supplementary file1 (DOCX 279 KB)

## Data Availability

The datasets are included in this article and available from the corresponding author on reasonable request.
