# Supplementary material for: Transcriptional Knock-down of mstn Encoding Myostatin Improves Muscle Quality of Nile Tilapia (Oreochromis niloticus)
Source: Mar Biotechnol (NY). 2023 Sep 27;25(6):951–65. doi: 10.1007/s10126-023-10252-1 (PMC10748778; doi:10.1007/s10126-023-10252-1)
Supplement: Supplementary file 1 — Supplementary file1 (DOCX 279 KB) [file 10126_2023_10252_MOESM1_ESM.docx]

**Supplementary Material**

1. Supplementary Figures

**
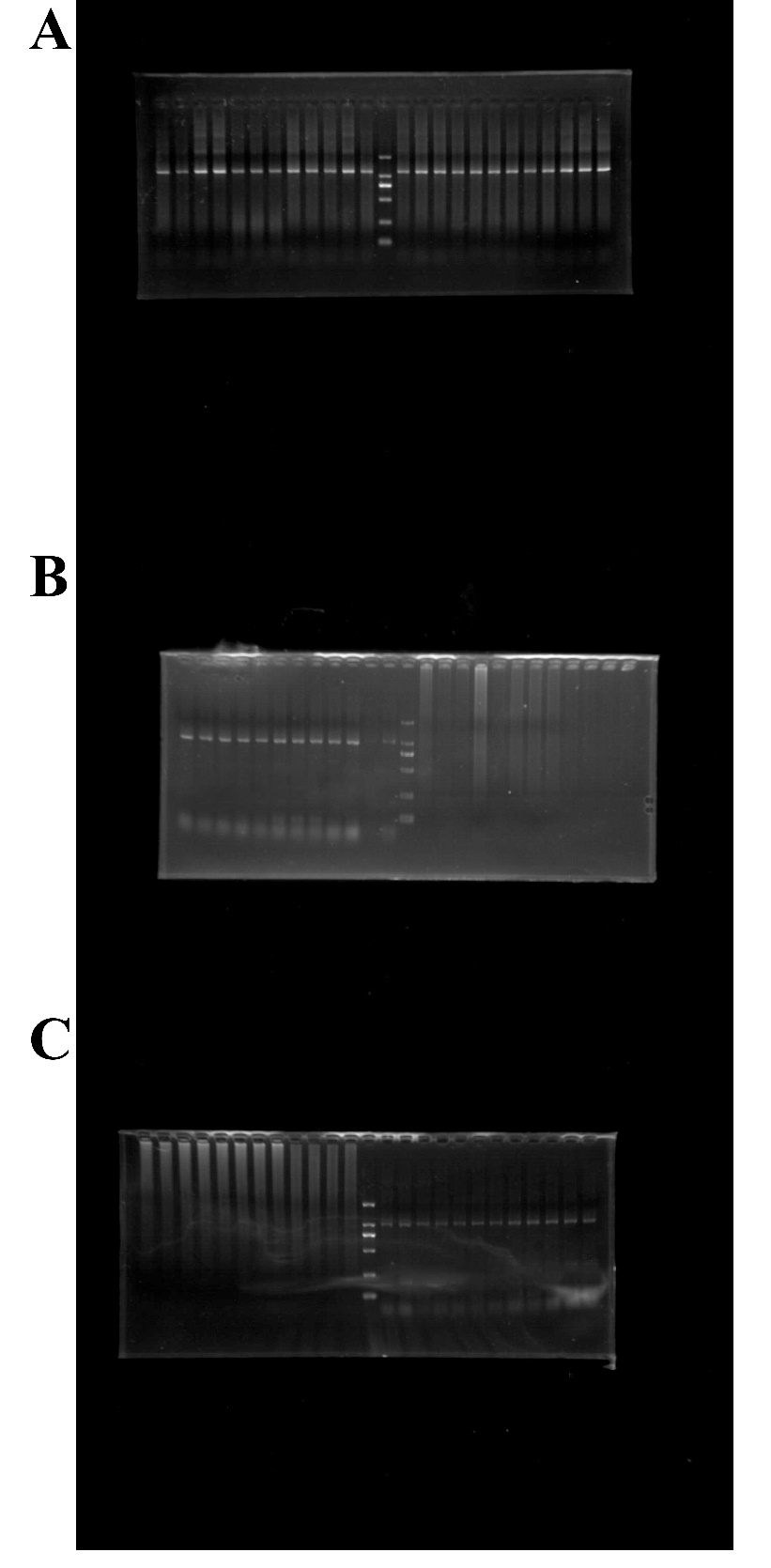
**

**Figure S1.** Detection of positive transfection rate by analysis of muscle tissue. (A) PCR analysis of muscle tissue from treatment group transfected with antisense RNA fragment, with an obvious band at about 1100 bp (plasmid 1000 bp + antisense RNA fragment of about 100 bp). A1–A12 (red box) represent 12 replicates from treatment group; (B) PCR analysis of muscle tissue from negative control (NC) group with a band of 1000 bp (plasmid 1000 bp). B1–B12 (red box) represent 12 replicates; (C) PCR analysis of muscle tissue from control group, with no obvious band at the position of 1000–1100 bp. C1–C12 represent 12 replicates.


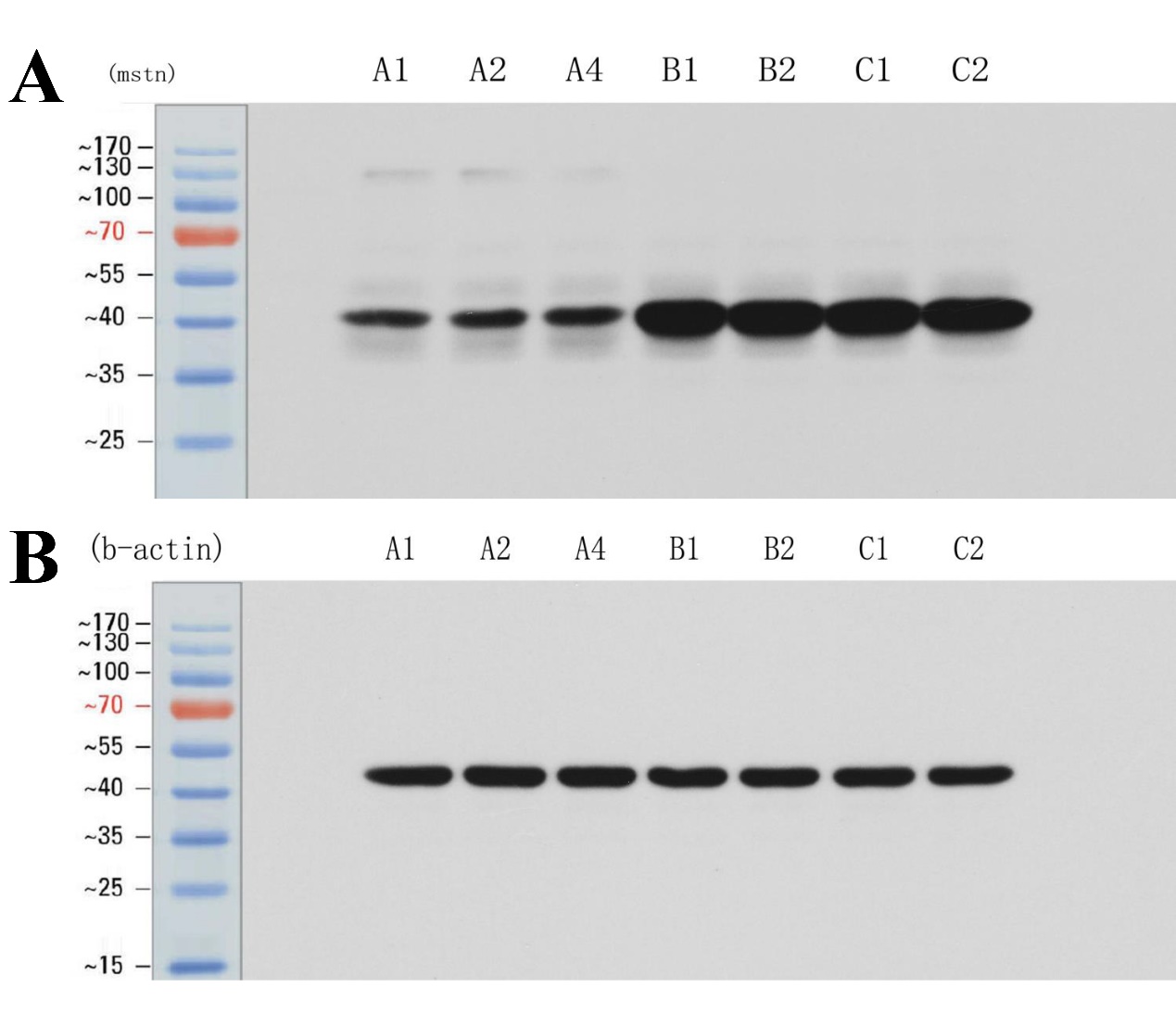


**Figure S2.** Western blot analyses showing relative myostatin (*mstn*) levels (A) in muscle tissue in treatment and control groups. β-actin (B) was the internal reference in each group.
